# Variability in antroduodenal and colonic manometry protocols across pediatric centers worldwide

**DOI:** 10.1038/s41390-025-04042-9

**Published:** 2025-04-28

**Authors:** Lev Dorfman, Khalil El-Chammas, Lin Fei, Ajay Kaul

**Affiliations:** 1 Gastroenterology, Hepatology and Nutrition, Cincinnati Children’s Hospital Medical Center, Cincinnati, OH USA; 2 School of Medicine, Faculty of Medical and Health Sciences, Tel Aviv University, Tel Aviv, Israel; 3 Institute of Gastroenterology, Nutrition and Liver Diseases, Schneider Children’s Medical Center of Israel, Petach Tikva, Israel; 4 Department of Pediatrics, University of Cincinnati, College of Medicine, Cincinnati, OH USA; 5 Biostatistics, Cincinnati Children’s Hospital Medical Center, Cincinnati, OH USA

## Abstract

**Background:**

Antroduodenal (ADM) and colonic (CM) manometries are performed in pediatric patients to evaluate gastrointestinal motility disorders. While minimal standards for performing ADM and CM have been published, there are no standardized protocols for performance of these studies.

We aimed to evaluate the diversity in ADM and CM protocols in pediatric centers worldwide.

**Methods:**

A cross-sectional study using an anonymous survey was conducted among pediatric centers worldwide comparing US and non-US centers.

**Results:**

Responses were received from 37 pediatric centers. ADM: 17 (45.9%) centers perform next-day and 14 (37.8%) centers perform same-day studies. Study length ranges from 4 to 24 hours. Erythromycin stimulation is implemented by 29/33 (87.9%), azithromycin by 15/33 (45.5%) and octreotide by 7/33 (21.2%) centers. US centers perform more pharmacological stimulations (30/31 (96.8%) vs. 3/6 (50%), *p* = 0.0018), while non-US centers conduct longer studies (15.3 hours vs. 7.4 hours, *p* = 0.0291).

CM: 17 (45.9%) centers perform same-day studies with length from 4 to 24 hours, and 89.2% perform pharmacological stimulation, all using bisacodyl.

**Conclusions:**

Significant variability exists in ADM and CM protocols among pediatric centers, affecting study length and pharmacological stimulation. Universal standardized guidelines are needed to ensure uniformity in the performance and interpretation of these studies.

**Impact:**

- Pediatric gastroenterological societies set minimal standards for antroduodenal (ADM) and colonic (CM) manometry studies, allowing variability in timing, fasting, postprandial phases, and provocation tests.
- Protocol variability among pediatric centers worldwide has not been previously assessed.
- This study offers a real-life overview of ADM and CM practices in pediatric centers worldwide, highlighting the need for standardized guidelines due to observed variability in timing, duration, and pharmacologic stimulation.
- The lack of standardization affects study interpretation and underscores the importance of developing universal guidelines.

## Introduction

Motility disorders and disorders of gut-brain interactions (DGBI) have increased in prevalence over the last two decades; this is partly attributed to improved diagnostic criteria and advancements in diagnostic modalities.^1,2^ To address that need, special focus was placed on pediatric neurogastroenterology and motility (NGM) training, and many centers worldwide developed pediatric neurogastroenterology and motility programs.^3–7^

The spectrum of pediatric NGM centers varies from centers conducting outpatient investigations, including esophageal and anorectal manometry, to centers that perform more complicated studies such as antroduodenal (ADM) and colonic (CM) manometry, demanding additional resources and training.

ADM and CM studies require the placement of manometry catheters under general anesthesia followed by a recording that lasts several hours to more than a day.^8–10^ Minimal standards for performing pediatric motility studies were published initially in 2002, followed by a consensus document on pediatric ADM and CM studies by the North American Society for Pediatric Gastroenterology, Hepatology and Nutrition (NASPGHAN) and the American Neurogastroenterology and Motility Society (ANMS) addressing the preparation, equipment, conduction and interpretation of ADM and CM studies, published in 2016 and 2017.^11–13^ Recently, the European Society of Paediatric Gastroenterology, Hepatology and Nutrition (ESPGHAN) motility working group published a review presenting suggested CM investigation protocol.^14^

The variability in the performance of ADM and CM studies includes the type of catheter used (including water-perfused and solid state catheters) and method of placement, timing of manometry recording after manometry catheter placement, the length of fasting, postprandial phases, and the provocation tests used.^10,15–18^

Such variability not only impacts study duration, a crucial factor influencing study feasibility and the utilization of health resources, but also triggers doubts concerning the interpretation of the data collected.

Our objective was to evaluate the diversity in protocols utilized by pediatric motility centers globally in the performance of ADM and CM studies, aiming to collect real-world data with the potential to establish universal standards for performing ADM and CM studies in the future.

## Materials and Methods

We conducted a cross-sectional study based on a structured internet survey among pediatric neuro-gastroenterology centers worldwide. The survey was conducted from December 2023 to January 2024. Pediatric neurogastroenterology and motility centers outside of the US were approached through neurogastroenterology and motility societies worldwide, while pediatric neurogastroenterology and motility services in North America were approached using a list published by the North American Society of Pediatric Gastroenterology, Hepatology and Nutrition (NASPGHAN) Neurogastroenterology and motility (NGM) Committee in 2022.^5^

The survey questionnaire was designed specifically for this study and included 22 questions assessing the protocols followed during the performance of ADM and CM studies (Table 1). The survey was performed on Google Forms and distributed via e-mail among pediatric neurogastroenterology centers worldwide.

**Table 1:** Comparison of antroduodenal manometry protocol variables among US and non-US centers

Survey question	*N*	Survey answer / parameter	Non-US centers (*N* = 6)	US centers (*N* = 31)	Overall (N = 37)	*p* value
1. When is antroduodenal manometry study performed?	37	Next day of catheter placement, *n* (%)	2 (33.3%)	15 (48.4%)	17 (45.9%)	0.439
		Same day of catheter placement, *n* (%)	3 (50%)	11 (35.5%)	14 (37.8%)	
		Same day and repeated next day if abnormal, *n* (%)	0 (0%)	4 (12.9%)	4 (10.8%)	
		Both, *n* (%)	1 (16.7%)	1 (3.23%)	2 (5.4%)	
2. Which manometry catheters do you usually use?	37	Water perfused, *n* (%)	4 (66.6%)	5 (16.1%)	9 (24.3%)	**0.024**
		Solid state, *n* (%)	2 (33.3%)	14 (45.2%)	16 (43.2%)	
		Both, *n* (%)	0 (0%)	12 (38.7%)	12 (32.4%)	
3. What is the approximate total length of ADM study?	37	Hours, mean (SD)	15.3 (9.5)	7.4 (4.6)	8.7 (6.2)	**0.0291**
		Hours, median, (IQR)	16 (6, 24)	6 (6, 8)	6 (6, 8)	
4. What is the approximate length of the fasting phase during the ADM study?	37	Hours, mean (SD)	4.2 (2.2)	2.7 (0.87)	2.9 (1.3)	**0.0285**
		Hours, median, (IQR)	4 (4,4)	3 (2, 3)	3 (2,4)	
5. What is the approximate length of the post prandial phase during the ADM study?	37	Hours, mean (SD)	2 (1.1)	1.4 (0.56)	1.5 (0.69)	0.15
		Hours, median, (IQR)	2 (1,2)	1 (1,2)	1 (1,2)	
6. Do you perform pharmacological stimulation during the ADM study?	37	Always, *n* (%)	1 (16.7%)	26 (83.9%)	17 (45.9%)	**0.0018**
		As needed, *n* (%)	2 (33.3%)	4 (12.9%)	14 (37.8%)	
		Never, *n* (%)	3 (50%)	1 (3.23%)	4 (10.8%)	
7. Which medication do you use for gastric stimulation during ADM study?	33	Erythromycin, *n* (%)	3 (100%)	26 (86.7%)	29 (87.9%)	1
		Azithromycin, *n* (%)	0 (0%)	17 (56.7%)	17 (51.5%)	0.1
		Octreotide, *n* (%)	0 (0%)	7 (23.3%)	7 (21.2%)	1
8. What is the usual length of gastric post stimulation phase?	33	Hours, mean (SD)	1.5 (0.5)	1.1 (0.3)	1.14 (0.34)	**0.0499**
		Hours, median, (IQR)	1.5 (1,2)	1 (1,1)	1 (1,1)	
9. Do you repeat stimulation in case of lack of gastric response to first stimulation?	33	Yes, *n* (%)	1 (33.3%)	13 (43.3%)	14 (42.4%)	1
10. Which medication do you usually use for duodenal stimulation during antroduodenal manometry study?	33	Octreotide, *n* (%)	3 (100%)	27 (90%)	30 (90.9%)	1
		Erythromycin, *n* (%)	1 (33.3%)	10 (33.3%)	11 (33.3%)	1
		Azithromycin, *n* (%)	0 (0%)	5 (16.7%)	5 (15.2%)	1
		Amoxicillin-clavulanate, *n* (%)	0 (0%)	1 (3.3%)	1 (3.03%)	1
11. What is the usual length of duodenal post stimulation phase?	33	Hours, mean (SD)	1.5 (0.5)	1.1 (0.3)	1.14 (0.34)	**0.0499**
		Hours, median, (IQR)	1.5 (1,2)	1 (1,1)	1 (1,1)	
12. Do you repeat stimulation in case of lack of duodenal response to first stimulation?	33	Yes, *n* (%)	0 (0%)	14 (46.7%)	14 (42.4%)	0.244

Statistically significant *p*-values are in bold

A comparison was performed between US centers and the rest of the world. Statistical analysis included frequency and percentages to summarize the survey answers. Fisher’s exact test was used to compare differences of categorical responses in the survey answers by the group. Wilcoxon test was used to compare differences of numerical responses.

The study was reviewed by the Cincinnati Children’s Hospital Institutional Review Board (IRB) and determined to be exempt from IRB review in accordance with applicable regulations and institutional policy due to its anonymous design (IRB number 2023-0714).

## Results

We received responses from 37 pediatric centers worldwide. All participants completed the entire survey.

**Fig. 1:**
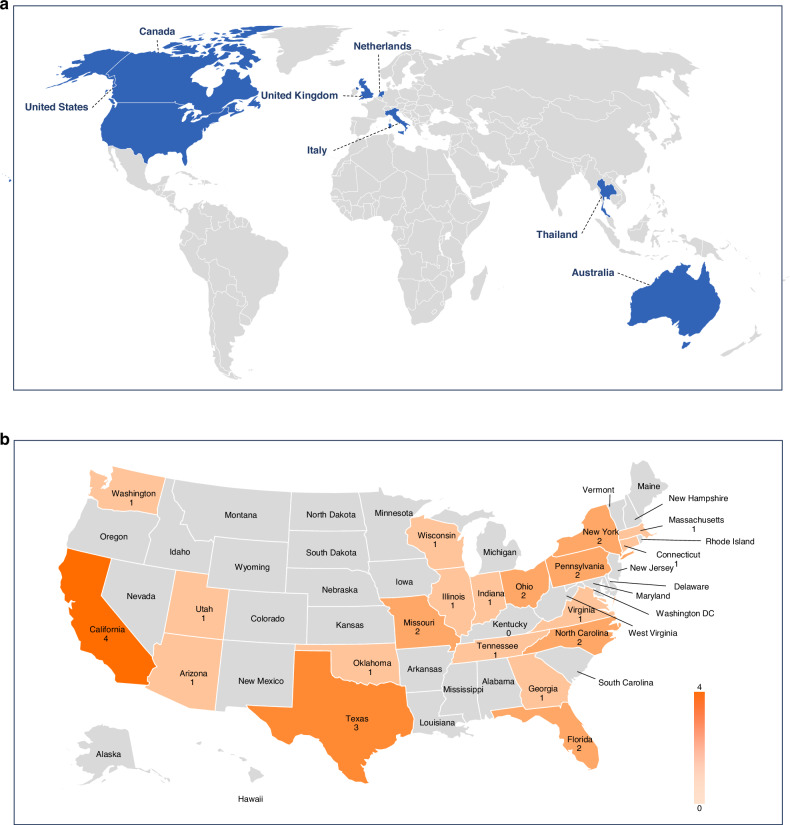
Survey participants. **a**. Participating centers worldwide. **b**. Participating centers across the US.

The majority (31, 83.8%) were from the USA including centers from a total of 21 different states, while an additional 6 were from Canada, the United Kingdom, the Netherlands, Italy, Thailand, and Australia (Fig. 1).

### Antroduodenal manometry

Solid-state manometry catheters are solely used by 16 (43.2%) centers, water perfused manometry catheters are solely used by 9 (24.3%) centers and 12 (32.4%) centers use both.

A total of 17 (45.9%) centers perform ADM studies only the next day after manometry catheter placement, while 14 (37.8%) centers perform only same-day manometry studies; 4 (10.8%) centers perform a same-day study that is continued the next day study only if abnormal results are noted on the first day, and 2 (5.4%) of centers perform ADM recordings during both same day and next day of placement, regardless of the results of the same day testing.

**Fig. 2:**
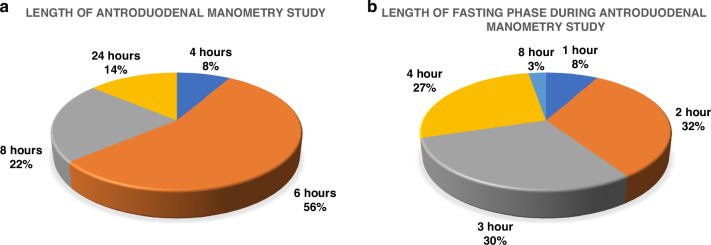
Heterogeneity in pediatric Antroduodenal manometry protocols worldwide. **a** Variability in length of antroduodenal studies worldwide. **b** Variability in length of the fasting phase during colonic manometry studies worldwide.

The length of the study ranged from 4 to 24 hours, with 3 (8.1%) of centers performing a study of 4-hour long, 21 (56.8%) centers performing a 6-hour long study, 8 (21.6%) centers performing 8-hour long study, and 5 (13.5%) centers performing a 24-hour long study (see Fig. 2). The length of the fasting phase lasts for 1 hour in 3 centers, 2 hours in 12 centers, 3 hours in 11 centers, 4 hours in 10 centers, and 8 hours in 1 center. The postprandial phase is studied for 1 hour in 21 (56.8%) centers, followed by a 2-hour postprandial period implemented by 14 (37.8%) centers, 3 hour postprandial phase reported by one center (2.7%), and a 4 hour postprandial phase reported by another center (2.7%). One center performing the study over both days provides 2 meals during the study period.

Pharmacologic stimulation is always performed during ADM by 27 (73%) centers, while 6 (16.2%) centers perform pharmacologic stimulation as needed, and 4 (10.8%) centers never perform pharmacological stimulation. Among the centers that perform pharmacological stimulation, erythromycin was the most popular agent for gastric stimulation, implemented by 29/33 (87.9%) centers, followed by azithromycin used for stimulation by 15/33 (45.5%) centers. Octreotide was reported to be used to provoke gastric contraction by 7/33 (21.2%) centers. Repeating gastric stimulation in case of lack of gastric response is not performed by 19/33 (57.6%) centers, while the rest 14/33 (42.4%) centers perform repeated gastric stimulation if needed.

For stimulation of migrating motor complexes (MMC) in the duodenum, octreotide is used by 30/33 (90.9%) centers, erythromycin by 11/33 (33%) centers, azithromycin by 5/33 (15.2%) centers, and amoxicillin-clavulanic acid by 1/33 (3%) centers. Repeat duodenal MMC stimulation in case of lack of duodenal response is not performed by 19/33 (57.6%) centers while the rest 14/33 (42.4%) perform repeat duodenal stimulation of MMC if needed.

Two centers reported repeating gastric but not duodenal stimulation, while two centers reported that they repeat only the duodenal phase if needed.

The length of post-stimulation recording was reported to be half an hour in one center (3%), 1 hour in 25 (75.8%) centers, 1.5 hours in 4 (12.1%) centers, and 2 hours in 3 (9.1%) centers. The lengths were reported to be similar for postgastric and postduodenal stimulation.

When comparing US and non-US centers, significantly more US centers use both solid state and water perfused catheters (12/31(38.7%) while none of the non-US centers use water perfused and solid state manometry within the same institution (*p* = 0.024). In addition, more US centers perform pharmacological stimulation during ADM studies compared to non-US centers (30/31 (96.8%) vs. 3/6 (50%), *p* = 0.0018).

Variability in the length of recording of ADM study was noted among both US and non-US centers, with study length ranging from 6 to 24 hours in non-US centers and from 4 to 24 hours in US centers. The length of recording of fasting phase ranged from 1 to 8 hours in the non-US centers and from 1 to 4 hours in the US centers. Length of recording of both gastric and duodenal post-stimulation phases ranged from 1 to 2 hours in the non-US centers and from 0.5 to 2 hours in the US centers.

The total length of ADM study recording was significantly longer in the non-US centers (average of 15.3 hours vs. 7.4 hours, p = 0.0291). Furthermore, the length of recording of the fasting phase was longer among the non-US centers with an average length of 4.2 hours compared to 2.7 hours in the US centers (*p* = 0.0285). In addition, there was a difference in the length of recording of the post-stimulation phase, which was significantly longer among the non-US centers (1.5 hours vs. 1.1 hours, p = 0.0499). The summary of the AD manometry study comparison is presented in Table 1.

### Colonic manometry

Solid state CM catheters are solely used by 18 (48.7%) centers, water-perfused manometry catheters are solely used by 7 (18.9%) centers, and 12 (32.4%) centers use both methods.

A total of 14 (37.8%) centers perform CM studies the next day after manometry catheter placement, while 17 (45.9%) centers perform same-day manometry studies. The same 4 (10.8%) centers that perform ADM studies on the same day of catheter placement and convert to a next-day study if abnormal results are noted use the same protocol for CM studies as well.

Two centers (5.4%) perform CM recordings both on the same day as well as on the next day of placement, regardless of the results of the same-day recording.

The total length of CM study recording is 4 hours in 8 (21.6%) centers, 6 hours in 22 (%) centers, 8 hours in 5 (13.5%) centers, and 24 hours in 2 (5.4%) centers. The length of recording of the fasting phase is 1 hour in 11 (29.7%) centers, 2 hours in 15 (40.5%) centers, 2.5 hours in 1 (2.7%) center, 3 hours in 6 (16.2%) centers, 4 hours in 3 (8.1%) centers, and 8 hours in 1 (2.7%) center (Fig. 2).

The length of recording of the postprandial phase was reported to be 1 hour by 22 (59.5%) centers, 1.5 hour by 1 (2.7%) center, 2 hours by 12 (32.4%) centers, and 3 hours by 2 (5.4%) centers.

The majority of 33 (89.2%) centers always perform pharmacological stimulation during CM studies, 3 (8.1%) centers perform stimulation as needed, and 1 (2.7%) center never performs pharmacological stimulation during CM. All centers use bisacodyl for pharmacological stimulation during CM studies, while 2 (5.6%) centers also use glycerin, 1 (2.8%) center also uses senna and 1 (2.8%) center also uses prucalopride for colonic stimulation. Repeated stimulation with bisacodyl is performed in case of lack of colonic response by 34 (94.4%) centers. The length of recording of post post-stimulation phase lasts 30 minutes in 1 (2.8%) center, 1 hour in 24 (66.7%) centers, 1.5 hours in 1 (2.8%) center, 2 hours in 9 (25%), and 3 hours in 1 (2.8%) center.

Comparing non-US and US centers, significantly more US centers reported using both solid state and water perfused catheters in the same center (12/31 (38.7%) while all non-US centers use just one type of catheter in the same center 0 (0, 0%), p = 0.0438).

Variability in the performance of CM study length was noted among both US and non-US centers with length of recording ranging from 4 to 8 hours in the non-US centers and from 4 to 24 hours in the US centers. The length of recording of fasting phase ranged from 1 to 3 hours in non-US centers and from 1 to 8 hours in the US centers. The length of recording of post-stimulation phase in CM ranged from 1 to 3 hours both in US and non-US centers.

No other differences were noted among non-US and US centers in the performance of CM study protocols. Comparison in the performance of CM protocols in US and non-US centers is presented in Table 2.

**Table 2:** Comparison of colonic manometry protocol variables among US and non-US centers

Survey question	*N*	Survey answer / parameter	Non-US centers (*N* = 6)	US centers (*N* = 31)	Overall (*N* = 37)	*p* value
1. When is colonic manometry study performed?	37	Next day of catheter placement, *n* (%)	1 (16.7%)	13 (41.9%)	14 (37.8%)	0.223
		Same day of catheter placement, *n* (%)	4 (66.6%)	13 (41.9%)	17 (45.9%)	
		Same day and repeated next day if abnormal, *n* (%)	0 (0%)	4 (12.9%)	4 (10.8%)	
		Both, *n* (%)	1 (16.7%)	1 (3.23%)	2 (5.4%)	
2. Which manometry catheters do you usually use?	37	Water perfused, *n* (%)	3 (50%)	4 (12.9%)	7 (18.9%)	**0.024**
		Solid state, *n* (%)	3 (50%)	15 (48.4%)	18 (48.6%)	
		Both, *n* (%)	0 (0%)	12 (32.4%)	12 (32.4%)	
3. What is the approximate total length of CM study?	37	Hours, mean (SD)	6 (1.8)	6.97 (4.7)	6.81 (4.3)	1
		Hours, median, (IQR)	6 (4, 8)	6 (6, 6)	6 (6, 6)	
4. What is the approximate length of the fasting phase during the CM study?	37	Hours, mean (SD)	1.66 (0.8)	2.3 (1.4)	2.2 (1.3)	0.233
		Hours, median, (IQR)	1.5 (1,2)	2 (1, 3)	2 (1,3)	
5. What is the approximate length of the post prandial phase during the CM study?	37	Hours, mean (SD)	1.8 (0.8)	1.37 (0.5)	1.45 (0.6)	0.1
		Hours, median, (IQR)	2 (1,2)	1 (1,2)	1 (1,2)	
6. Do you perform pharmacological stimulation during the CM study?	37	Always, *n* (%)	5 (83.3%)	28 (90.3%)	33 (89.2%)	0.523
		As needed, *n* (%)	1 (16.7%)	2 (6.45%)	3 (8.1%)	
		Never, *n* (%)	0 (0%)	1 (3.23%)	1 (2.7%)	
7. Which medication do you use for colonic stimulation during CM study?	36	Bisacodyl, *n* (%)	6 (100%)	30 (100%)	36 (100%)	1
		Glycerin, *n* (%)	0 (0%)	2 (6.67%)	2 (5.56%)	1
		Prucalopride, *n* (%)	0 (0%)	1 (3.33%)	1 (2.78%)	1
		Senna, *n* (%)	0 (0%)	1 (3.33%)	1 (2.78%)	1
8. What is the usual length of colonic post stimulation phase?	36	Hours, mean (SD)	1.3 (0.5)	1.3 (0.6)	1.3 (0.5)	0.798
		Hours, median, (IQR)	1 (1,2)	1 (1,2)	1 (1,2)	
9. Do you repeat stimulation in case of lack of colonic response to first stimulation?	36	Yes, *n* (%)	5 (83.3%)	29 (96.7%)	34 (94.4%)	0.309

Statistically significant *p*-values are in bold

## Discussion

This is the first study to evaluate the current clinical practice in conducting ADM and CM studies in pediatric centers worldwide.

### Study timing

ADM and CM studies are time-consuming, and the effect of anesthesia on these studies is a major debate among providers. Same-day studies shorten inpatient stays, reduce the risk of catheter dislodgment, and increase study feasibility. While one previous study showed that anesthesia did not affect CM findings, a later larger cohort showed that it significantly increased the rate of abnormal studies, which were interpreted as normal when repeated on the following day.^16,17^ Our survey reflects this clinical dilemma as 38% of the centers perform next-day CM manometry studies and 46% perform next–day ADM manometry studies, while another 46% perform same-day CM studies and 38% perform same-day ADM studies. This variability in practice affects the reliability and interpretation of manometry studies, especially due to previously published doubts regarding the anesthesia effect. Furthermore, the validity of abnormal findings noted in a study that was conducted on the same day after anesthesia is questionable. The combined same-day to next-day strategy is implemented by 4 (10.8%) centers in ADM and CM studies; this protocol takes advantage of both strategies, with an option to conclude the study if normal findings are noted on day one. While this strategy has the potential to shorten overall hospital stay, it requires more specialized staffing to perform manometry recording in the evening and may add additional risks to the patients, including fluid overload if run overnight. In addition, centers conducting studies with this combined, conditional approach have valuable data that, by retrospective and prospective evaluation in a multi-center large cohort of patients, can provide a better understanding of the effect of anesthesia on ADM and CM. Such data will support a universal standardized approach that can be implemented in international consensus guidelines and adopted among pediatric centers worldwide. In addition, a cost analysis should be performed to assess the cost-effectiveness of that strategy.

### Antroduodenal manometry

Great variability of the recording of ADM studies was noted in our survey with significantly longer studies (almost double on average) being conducted outside of the USA. While some centers stick to the minimal required standards for performing 6-hour long studies, others conduct studies that last 24 hours. While prolonged studies were shown to identify specific motor abnormalities in adult patients, study duration not only affects healthcare costs and reduces feasibility (especially in small children and children with developmental delay or behavioral disorders), but it also increases potential side effects in centers conducting studies with water-perfused catheters.^19^ In addition, Prolonged perfusion was shown to be a risk for electrolyte disturbance and prompts close electrolyte monitoring.^20^ Further research is needed to determine the optimal length of manometry studies in pediatric patients, aiming to maximize diagnostic value while minimizing the risk of adverse events and optimizing healthcare cost.

The main finding that reflects intact neuromuscular integrity on ADM is the presence of motor migrating complex III (MMC III), which is noted during the fasting phase.^9^ Accordingly, longer recording in the fasting period increases the chance to capture a MMC III. While in some centers, the recording of fasting period during ADM study lasts 1 hour, others record for up to 8 hours. No studies have been conducted to assess the optimal length of recording of fasting period during ADM studies. Such variability was noted to a lesser extent in the length of recording of the postprandial period (range of 1 to 4 hours).

The presence of phase III MMC in either fasting phase or after provocation with a pharmacologic agent during the ADM study, is interpreted as normal. Pharmacological agents to stimulate MMC can potentially shorten or replace the fasting phase. Duodenal provocation was reported to be performed mainly with octreotide – a protocol implemented by >90% of the centers. Erythromycin was also used by one-third of pediatric centers worldwide, with one center reporting the use of amoxicillin-clavulanate. Amoxicillin-clavulanate was previously shown to induce phase III MMC in a small cohort of patients, but further studies are needed to confirm this effect.^21^ Using different agents with different half-life and potency, which has not been previously compared in head-to-head studies, limits the interpretation of ADM studies. It should be noted that pharmacological stimulation is not performed by more than 10% of centers due to reasons that were not assessed in our study.

For gastric stimulation, most centers use erythromycin or azithromycin, with a minority using octreotide. Erythromycin was previously shown as an agent that can provoke MMC, but the rate of erythromycin-induced MMC in pediatrics has not been reported.^22^ Octreotide was reported to induce gastric motor activity in 76% of pediatric patients.^23^ A stepwise approach, using octreotide as the first agent that has the potential to provoke both gastric and duodenal activity, may spare the need to use an additional pharmacologic agent for gastric stimulation in a large subset of patients.

Repeating pharmacological stimulation is not part of the current ADM guidelines, but it is implemented by 42% of the centers for both gastric and duodenal provocation. The effect of repeated stimulation in studies with lack of gastric or duodenal response, has not been previously described, and further studies will help in determining the need for repeated stimulation.

### Colonic manometry

CM studies were reported to be shorter than ADM, with an average total length of 6.8 hours and without significant difference between the US and non-US centers. The range of length of recording of the fasting phase was reported from 1 to 8 hours with an average length of 2.2 hours. High amplitude propagated contractions (HAPC) are the hallmark sign of a normal CM study and can occur both during the fasting as well as the postprandial phases. Like for ADM studies, shortening the length or omitting the fasting phase during a CM study can be adopted with the use of pharmacologic stimulation if an HAPC is not noted in the postprandial phase^24^. Pharmacologic stimulation during a CM study is always performed in 89% of centers. An additional 8% reported using provocative agents only if no HAPC is noted during prior phases. This latter approach may reduce the total length of CM study and avoid the need for colonic stimulants, with potential side effects of abdominal cramping. It does, however, necessitate constant survey of the CM tracings to make the decision if a stimulant is needed.

Bisacodyl was reported to be the agent used for colonic stimulation by all centers, with an additional 5.5% percent of centers reporting the use of either bisacodyl or glycerin. Glycerin was shown to be inferior to bisacodyl in a recent pediatric prospective, single-center study using a cross-over design.^25^ One center in US reported using prucalopride and senna as the provocative agents, in addition to bisacodyl. The effect of prucalopride or senna on CM findings, has not been previously described.

Repeating stimulation with bisacodyl, which was previously shown to be beneficial for the exclusion of dysmotility during CM studies,^26^ was reported to be implemented by the majority of centers ( > 94%). The length of post-stimulation CM recording was reported over a wide range, from 30 minutes up to 3 hours.

When assessing the difference between the US and non-US centers, US centers more frequently utilize both solid-state and water-perfused catheters, whereas none of the non-US centers employ both systems within the same institution. Most non-US centers rely on the less expensive water-perfused systems, likely due to financial constraints.

Additionally, pharmacological stimulation during ADM studies is performed more commonly in US centers compared to non-US centers (30/31, 96.8%, vs. 3/6, 50%; *p* = 0.0018). The reasons for this difference are unclear but may involve cost considerations, limited access to pharmacological agents, or a preference for simpler protocols with fewer steps.

Interestingly, the total duration of ADM recordings was significantly longer in non-US centers, possibly as a compensatory approach for not conducting provocative tests. Overall, a noteworthy variability was noted among ADM and CM studies conducted in pediatric centers globally. This diversity was noted in the timing of study (same day vs. next day), study length, agents used for stimulation, and repeating stimulation. Currently published guidelines address the minimum standards for performing ADM and CM, allowing for a significant variability among different centers, which is reflected in our study. In adults, the Chicago classification establishes a standardized universal protocol for conducting esophageal manometry studies, and the London classification sets a standard procedure for anorectal manometry studies. However, there is currently a lack of similar standardization for ADM and CM studies both in adults and pediatrics.^27,28^

Our study is limited by its anonymous survey design, which lacks the ability to assess the reliability of the responses. Moreover, most centers are located in the US, with non-US centers being underrepresented. The predominance of US centers in our study may reflect more specialized pediatric NGM centers in the US relative to the rest of the world, larger US pediatric neurogastroenterology society, or a lower response rate among centers worldwide. There is no international global registry that comprehensively lists all pediatric centers performing ADM and CM, which could be utilized as a resource. This could lead to a selection bias in our manuscript. In addition, our survey did not cover all aspects of manometry procedures, such as placement techniques, the use of endoclips, sedation protocols, or performance with or without prokinetic medications. These aspects should be further investigated and addressed in the future development of a standardized universal protocol by an international experts working group.

ADM and CM are conducted in highly specialized centers by trained pediatric neurogastroenterologists. These studies have many indications, offering both diagnostic and therapeutic benefits. ADM findings have been shown to guide therapeutic approaches in pediatric patients with pseudo-obstruction, while CM has demonstrated value in decision-making prior to colonic resections or the reconnection of diverting ileostomies.^9,12,14,29^ Additionally, CM plays a crucial role in evaluating symptomatic patients with Hirschsprung disease.^30^ Standardizing procedures, reducing the duration of these studies, and expanding neurogastroenterology training will be pivotal in increasing their accessibility and widespread adoption.

## Conclusion

Our study reveals a great variability in the protocols in the performance of ADM and CM studies in children worldwide. The absence of standardized protocols contributes to greater variability in the execution and interpretation of ADM and CM studies. Given the time and resources involved in conducting these studies, particularly in the pediatric population, establishing a standardized protocol is an important goal that should be prioritized by experts and professional organizations

## Data Availability

The datasets generated during and/or analyzed during the current study are available from the corresponding author on reasonable request
